# Whole blood GBP5 protein levels in patients with and without active tuberculosis

**DOI:** 10.1186/s12879-022-07214-8

**Published:** 2022-04-03

**Authors:** Xiangyang Yao, Wei Liu, Xiaofei Li, Chenxi Deng, Tingdong Li, Zhouyue Zhong, Shuping Chen, Zhitan Ge, Xuejie Zhang, Shiyin Zhang, Yingbin Wang, Yongliang Liu, Chao Zheng, Shengxiang Ge, Ningshao Xia

**Affiliations:** 1 Xinglin Branch of the First Affiliated Hospital of Xiamen University, Xiamen, Fujian 361102 People’s Republic of China; 2 State Key Laboratory of Molecular Vaccinology and Molecular Diagnostics, National Institute of Diagnostics and Vaccine Development in Infectious Disease, Collaborative Innovation Centers of Biological Products, School of Public Health, Xiamen University, South Xiang’an Road, 361102 Xiamen, People’s Republic of China; 3 Third People’s Hospital of Kunming City, Kunming, Yunnan 650041 People’s Republic of China

**Keywords:** Guanylate binding protein 5, Whole blood, Protein level, Active tuberculosis, Interferon-gamma release assay

## Abstract

**Background:**

The host blood transcriptional levels of several genes, such as *guanylate binding protein 5* (*GBP5*), have been reported as potential biomarkers for active tuberculosis (aTB) diagnosis. The aim of this study was to investigate whole blood GBP5 protein levels in aTB and non-tuberculosis patients.

**Methods:**

An in-house immunoassay for testing GBP5 protein levels in whole blood was developed, and suspected aTB patients were recruited. Whole blood samples were collected and tested at enrolment using interferon-gamma release assay (IGRA) and the GBP5 assay.

**Results:**

A total of 470 participants were enrolled, and 232 and 238 patients were finally diagnosed with aTB and non-TB, respectively. The GBP5 protein levels of aTB patients were significantly higher than those of non-tuberculosis patients (p < 0.001), and the area under the ROC curve of the GBP5 assay for aTB diagnosis was 0.76. The reactivity of the GBP5 assay between pulmonary and extrapulmonary tuberculosis patients was comparable (p = 0.661). With the optimal cut-off value, the sensitivity and specificity of the GBP5 assay for diagnosing aTB were 78.02 and 66.81%, respectively, while those of IGRA were 77.59 and 76.47%. The combination of the GBP5 assay and IGRA results in 88.52% accuracy for diagnosing aTB in 63.83% of suspected patients with a positive predictive value of 89.57% and a negative predictive value of 87.59%.

**Conclusions:**

Whole blood GBP5 protein is a valuable biomarker for diagnosing of aTB. This study provides an important idea for realizing the clinical application of whole blood transcriptomics findings by immunological methods.

**Supplementary Information:**

The online version contains supplementary material available at 10.1186/s12879-022-07214-8.

## Introduction

Tuberculosis (TB) is one of the three major infectious diseases globally, with approximately 9.9 million new cases and 1.3 million deaths in 2020 [1]. Although the World Health Organization (WHO) and global researchers have made many efforts, TB remains a serious public health problem, particularly in developing countries [2]. The emergence of coinfection HIV/AIDS and drug-resistant TB cases makes TB control even more exigent [3, 4].

The rapid and accurate diagnosis of tuberculosis is essential for preventing of TB transmission. Several methods have been developed to diagnose TB; however, there are still challenges in the accurate diagnosis of smear negative pulmonary TB and extrapulmonary tuberculosis (EPTB) [5–8]. Pulmonary tuberculosis (PTB) is the most common clinical manifestation of active tuberculosis (aTB), which is usually diagnosed by the identification of MTB in sputum [8]. However, sputum smear microscopy has low sensitivity, and MTB culture is time-consuming (generally more than 2 weeks) [9, 10]. The nucleic acid detection of mycobacterium tuberculosis (MTB), such as GeneXpert MTB/RIF, is also widely used in the clinic, but its sensitivity in smear-negative sputum is still unsatisfactory [11, 12]. In addition, for these methods, it is challenging to collect diseased lesion samples from pulmonary TB patients who have no sputum and from extrapulmonary TB patients [13, 14]. TB antigen-specific interferon-gamma release assays (TB-IGRAs) have been introduced into clinical practice to diagnose MTB infection. For TB-IGRAs, heparinized whole peripheral blood is used as a sample, and its performance is independent of the sites where the diseased lesions are located. Unfortunately, IGRAs can only distinguish MTB infection from Bacillus Calmette-Guerin (BCG) vaccination and are unsuitable for discriminating aTB from latent tuberculosis infection (LTBI) [15, 16]. Many studies have shown that TB-IGRAs are not suitable for aTB diagnosis.

In 2016, by employing an integrated multicohort analysis of samples from a publicly available dataset, Sweeney et al. discovered a set of three genes, including *GBP5*, *DUSP3*, and *KLF2*, that are highly diagnostic for aTB [17]. Consistent with this finding, Francisco et al. reported that compared with those of *DUSP3* and *KLF2*, the transcriptional level of *GBP5* in whole blood had a significantly higher performance in discriminating aTB patients from healthy individuals and those with other lung diseases [18]. The above results indicate that the transcriptional level of *GBP5* in whole peripheral blood has the potential to be used as a biomarker for aTB diagnosis. This prompted us to investigate the difference in GBP5 protein levels in whole blood between aTB and non-aTB patients since immunoassays are more convenient than nucleic acid testing for clinical use.

In this study, we prepared mouse monoclonal antibodies against GBP5 and established an enzyme-linked immunosorbent assay (ELISA) for detecting GBP5 protein in whole blood. Then, we investigated the difference in GBP5 protein levels between aTB and non-tuberculosis (non-TB) patients. The results show that the GBP5 protein levels in whole blood are significantly higher in aTB than in non-TB, suggesting that the GBP5 protein level in whole blood is a potential biomarker for aTB diagnosis.

## Materials and methods

### Study participants

From February 2019 to May 2020, suspected aTB patients admitted to the Xinglin Branch of the First Affiliated Hospital of Xiamen University were recruited. The heparinized whole blood was collected from each recruited patient at enrolment for GBP5 protein detection and TB-IGRA. The diagnosis of aTB was based on clinical, radiological, microbiological, and histopathological information and the patient’s response to anti-TB therapy for at least 3 months, and the aTB patients included had PTB and EPTB individuals. For non-TB patients mainly include patients infected with non-tuberculosis mycobacterium (NTM) and other lung disease (OLD). In addition, the study counted the Immunosuppressive condition of participants, including HIV infection, chronic renal disease, diabetes, and underlying disease relevant for immunosuppressive treatment.

 The study was approved by the Ethical Committee of the School of Public Health, Xiamen University. Written informed consent was obtained from each participant.

### Preparation of GBP5 recombinant protein and anti-GBP5 antibodies

The coding sequence of GBP5 was synthesized by Sangon Biotech (Sangon Biotech Co., Ltd., Shanghai, China) and cloned into the expression vector pET-30a. Then, the recombinant plasmid was transformed into Escherichia coli BL21 (DE3). After induction by isopropyl-β-d-thiogalactoside, GBP5 was expressed in the form of inclusion bodies and further purified by Ni^2+^-NTA affinity chromatography under denaturing conditions. As analyzed by SDS-PAGE and HPLC, the purity of GBP5 was above 80% and the GBP5 protein was detected by western blot analysis using an anti-GBP5 rabbit pAb(Abcam, Cambridge, UK) (Additional file 1: Fig. S1). Balb/c mice were immunized with purified GBP5 (50 µg) emulsified in Freund’s adjuvant, and mouse monoclonal antibodies (mAbs) against GBP5 were prepared by hybridoma technology. A total of 70 mAbs against GBP5 were obtained and purified.

### Eukaryotic expression of GBP5

The synthesized cDNA of GBP5 was cloned into pTT5, a eukaryotic protein expression plasmid, and HEK 293 T cells were transfected for transient expression. At 48 h post transfection, cells were collected and lysed with lysis buffer (1% NP40 and 0.25% DOC in deionized water). After centrifugation, the supernatant was collected and stored at − 80 ℃ for mAb pair screening and ELISA establishment. The supernatants of HEK 293 T cells with transient expression of GBP5 and mock-transfected HEK 293 T cells were used as positive and negative controls, respectively.

### Double antibody sandwich ELISA

One mAb pair (7G9 and 9A9) with the highest positive to negative (P/N) ratio was selected to develop a double antibody sandwich ELISA to detect GBP5 protein in whole blood. mAbs 7G9 and 9A9 were employed for microplate coating and biotin conjugation, respectively, and the P/N ratio of these pairs exceeded 40 (Additional file 3: Fig. S2). The assay was developed and performed as below. In brief, 96-well polystyrene microplates (Xiamen Labware Co., Ltd., Xiamen, China) were coated with mAb 7G9 diluted with 20 mM phosphate buffer, pH 7.4, at a concentration of 4 µg/ml overnight at 37℃ followed by blocking with 200 µL of blocking buffer (2% BSA in 20 mM PBS) for 2 h. To detect GBP5 protein in whole blood, freshly collected heparinized whole peripheral blood was lysed with a fourfold volume of lysis buffer and centrifuged at 12,000 ×*g* for 10 min, and then the supernatant was added to the microplate. After incubation at 37 ℃ for 1 h, the microplate was washed 5 times with washing buffer (0.02% Tween-20 in 20 mM phosphate buffer saline, pH 7.4). Next, 100 µL of biotin-labelled mAb 9A9 (1 µg/ml in blocking buffer) was added to the washed microplate and incubated at 37 ℃ for 30 min, followed by another 5 washes. Then, 100 µL of horseradish peroxidase-conjugated streptavidin (Thermo Fisher Scientific, Rockford, IL) was added to the microplates and incubated at 37 ℃ for 30 min. After washing 5 times again, 100 µL of TMB substrate (Beijing Wantai, Beijing, China) was added and incubated at 37 ℃ for 15 min. Then, the colour development was stopped with 50 µL of 0.5 M sulfate, and the optical density (OD) at 450 nm with 630 nm as a reference was determined by an Autobio PHOmo microplate reader (Autobio, Zhengzhou, China).

### IGRA

According to the manufacturer’s instructions, the status of MTB infection was determined by WANTAI TB-IGRA (Beijing Wantai, Beijing, China) [19]. In brief, 1 ml of fresh heparinized venous whole blood was added into Eppendorf tubes containing nil for the negative control (N), mitogen for the positive control (P) and TB antigens (a recombinant protein of ESAT-6 and CFP-10) for the INF-γ test (T). After incubation at 37 °C for 22 ± 2 h, each tube was centrifuged, and the concentration of INF-γ in the plasma was measured. As recommended by the manufacturer, the cut-off of T-N was 14 pg/ml, and the results were valid if N ≤ 400 pg/ml and P-N ≥ 20 pg/ml simultaneously.

### Statistical analysis

Analyses were carried out with SPSS 25.0 (IBM Corp., Armonk, NY, USA). The Mann-Whitney U test or Fisher’s exact test was performed to compare the differences between two groups; the analyses were two-tailed, and statistical significance was considered when p <0.05. Receiver operating characteristic (ROC) analyses were performed by GraphPad Prism 8.0 (GraphPad Software, CA, USA) to evaluate the assay’s performance in distinguishing aTB patients from non-TB patients. The area under the curve (AUC) was calculated, and the optimum cut-off values were selected based on Youden’s index.

## Results

### Study population

From February to May 2019, 470 patients were enrolled in this study. Finally, 232 and 238 patients were diagnosed with aTB and non-TB, respectively. As shown in Table 1, the aTB patients were younger than the non-TB patients (median age 51 vs. 63.5, p < 0.001), and the aTB group had more male individuals (M/F 3.7 vs. 2.0, p = 0.003). The proportions of patients under immunosuppressive conditions in both groups were similar (14.66% vs. 12.61%), and aTB patients had a significantly higher IGRA positive rate (77.59%) than non-TB patients (23.53%) (p < 0.001). Among 232 aTB patients, 204 (87.93%) had PTB, and 79 (34.05%) had EPTB. There were 51 patients with PTB and EPTB simultaneously. For convenience in the next context, these 51 patients were included in the PTB group (n = 204), and the EPTB group only included EPTB patients without pulmonary manifestations (n = 28) (Table 2). Among 238 non-TB patients, 11 (4.62%) were infected by NTM, and 227 (95.38%) patients had OLD (Table 2).

**Table 1:** Demographic characteristics of the study population, n = 470

	aTB	non–TB	P value
Number of individuals	232	238	
Sex (male: female)	3.73 (183:49)	1.98 (158:80)	0.003
Age (median, range)	51.00 (14.00–89.00)	63.50 (4.00–91.00)	< 0.001
Immunosuppressive condition	34 (14.66%)	30 (12.61%)	0.591
HIV infection	3 (1.29%)	6 (2.52%)	0.504
Chronic renal disease	4 (1.72%)	3 (1.26%)	0.721
Diabetes	24 (10.34%)	16 (6.72%)	0.187
Underlying disease relevant for immunosuppressive treatment*	3 (1.29%)	5 (2.10%)	0.724
IGRA positive	180 (77.59%)	56 (23.53%)	< 0.001

*aTB* active tuberculosis, *non-TB* non-tuberculosis other lung diseases, *HIV* human immunodeficiency virus, *IGRA* interferon gamma release assay

*Includes rheumatologic disease, ankylosing spondylitis and systemic lupus erythematosus

**Table 2:** The comparison of reactivity between GBP5 and IGRA in different clinical groups

	n	GBP5+	IGRA+	P value
Active TB	232	181 (78.02%)	180 (77.59%)	>0.999
PTB	204	158 (77.45%)	160 (78.43%)	0.905
EPTB	28	23 (82.14%)	20 (71.43%)	0.528
non–TB	238	79 (33.19%)	56 (23.53%)	0.025
NTM	11	8 (72.73%)	2 (18.18%)	0.030
OLD*	227	71 (31.28%)	54 (23.79%)	0.093

The sensitivity, specificity, positive predictive value, and negative predictive value of the GBP5 assay were 78.02, 66.81, 69.61 and 75.71%, respectively, and those of IGRA were 77.59, 76.47%, 76.27 and 77.78%

*GBP5+* Individual with positive GBP5 result, *IGRA+* Individual with positive interferon gamma release assay result, *Active TB* active tuberculosis, *PTB* pulmonary tuberculosis, *EPTB* extrapulmonary tuberculosis, *non-TB* non-tuberculosis other lung diseases, *NTM* non-tuberculosis mycobacterium, *OLD* other lung disease

*Including fungal pneumonia (n = 7), mycoplasma pneumonia (n = 3), bacterial pneumonia (n = 173), lung cancer (n = 16) and non-infection non-cancer lung disease (n = 28)

### Levels of GBP5 protein in whole blood of aTB and non-TB

**Fig. 1:**
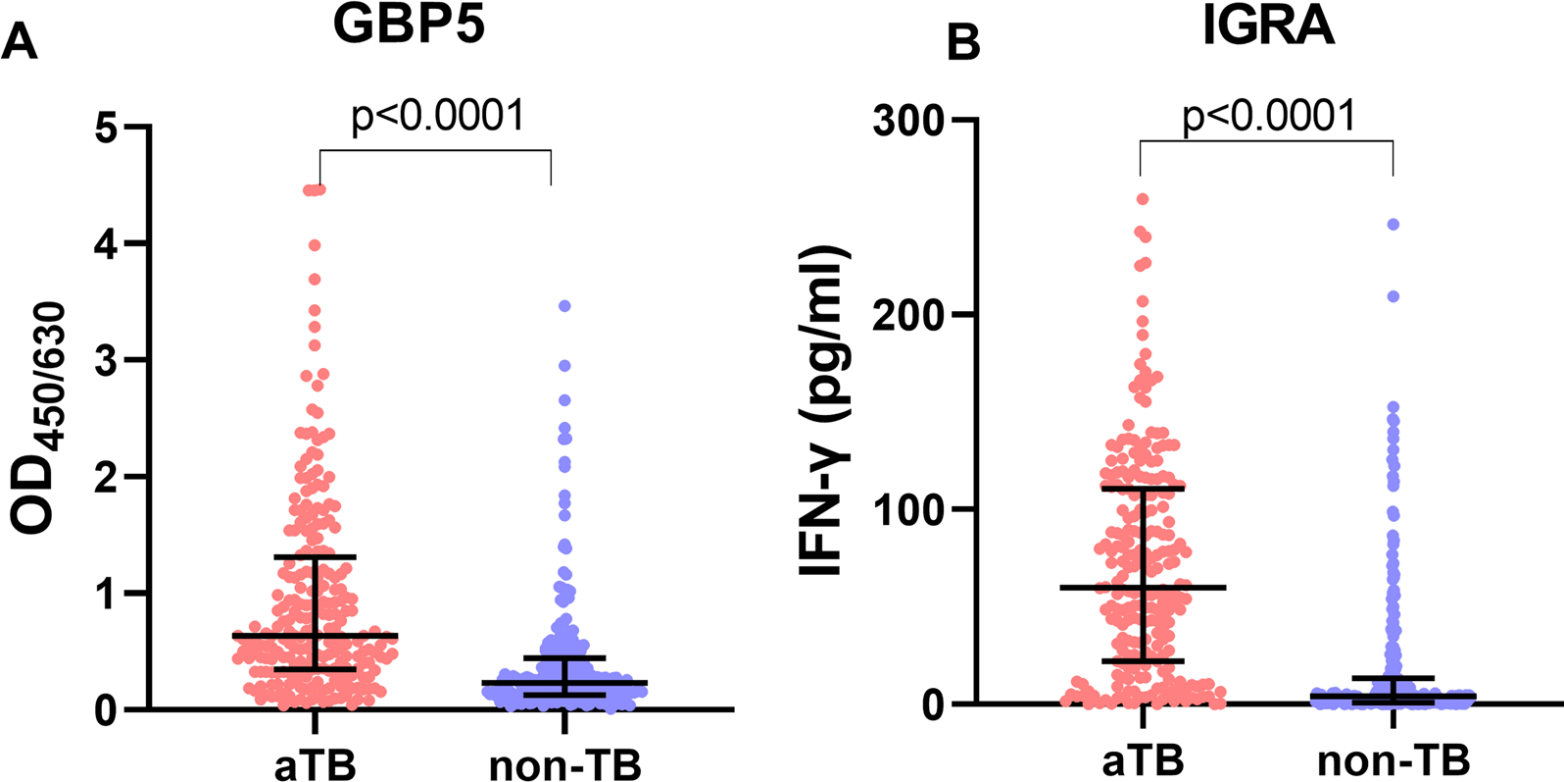
The levels of GBP5 protein and IGRA results between aTB and non-TB. **A** The levels of whole blood GBP5 protein levels in aTB and non-TB patients. **B** IGRA results (levels of IFN-γ response) in aTB and non-TB patients

**Fig. 2:**
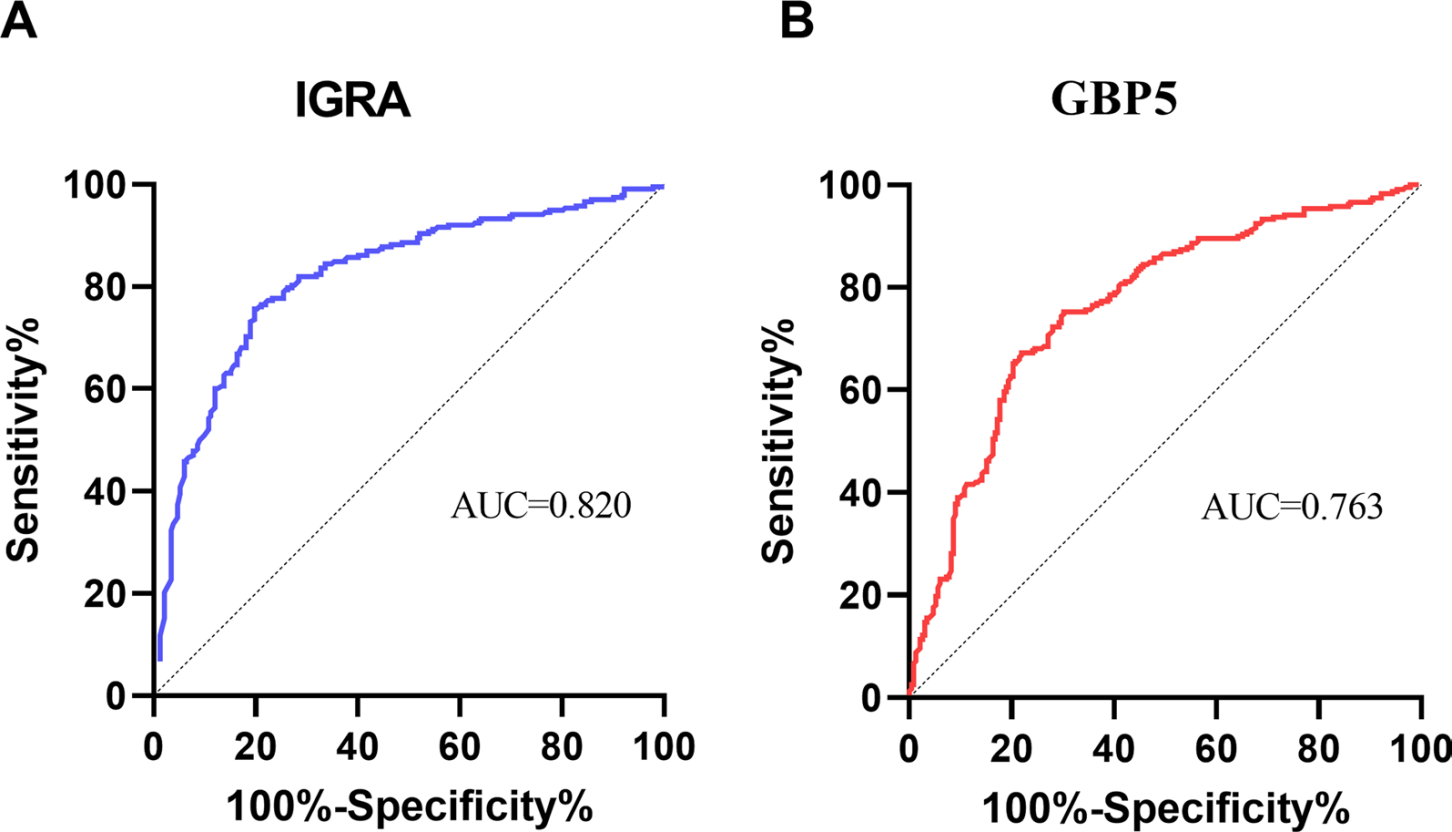
The levels of GBP5 protein and IGRA results in different aTB and non-TB patients. **A** The levels of whole blood GBP5 protein levels in PTB, EPTB, NTM and OLD patients. **B** IGRA results (levels of IFN-γ response) in PTB, EPTB, NTM and OLD patients. Bars represent the median and interquartile range

**Fig. 3:**
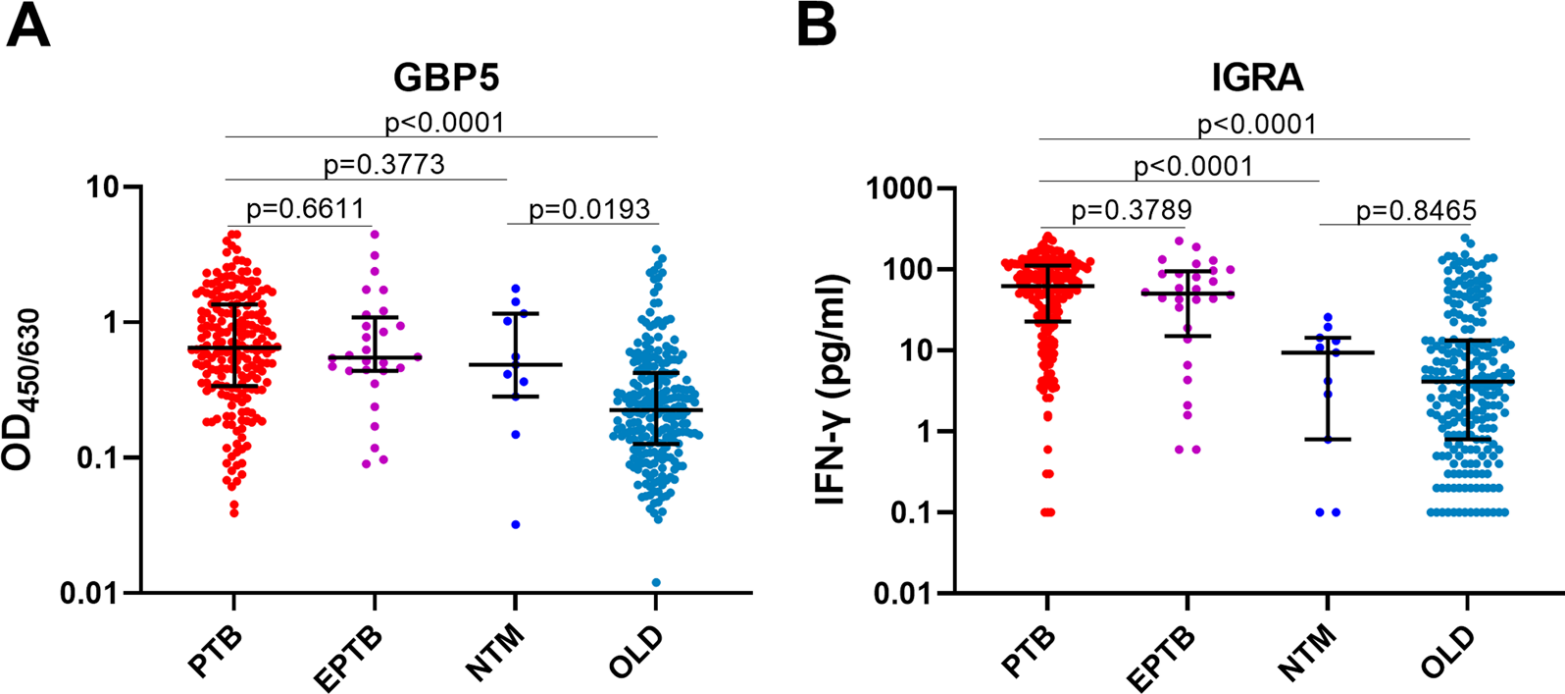
ROC curves of GBP5 and IGRA for the diagnosis of aTB. **A** IGRA. **B** GBP5. Bars represent the median and interquartile range

All participants were tested with whole blood GBP5 protein ELISA and TB-IGRA at enrolment. For both assays, the response of aTB was significantly higher than that of non-TB (p < 0.001) (Fig. 1A, B). The results indicate that aTB patients had elevated GBP5 protein expression in whole blood compared to non-TB patients. No significant difference in GBP5 response between PTB and EPTB was observed (p = 0.661), while GBP5 response in NTM was significantly higher than that in OLD (p = 0.019) (Fig. 2A). However, the IGRA responses in NTM and OLD were comparable (p = 0.847) (Fig. 2B). When ROC analysis was conducted, the area under the ROC curve (AUC) of the whole blood GBP5 protein assay (0.76) for diagnosing aTB was only slightly lower than that of IGRA (0.82) (p = 0.046) (Fig. 3A, B).

### The performance of whole blood GBP5 protein levels for aTB diagnosis

According to the ROC analysis, the optimal cut-off value of whole blood GBP5 was determined to be 0.324. With this cut-off value, the sensitivity of specificity of whole blood GBP5 protein in this cohort were 78.02% (181/232) and 66.81% (159/238), respectively (Table 2). Meanwhile, using the cut-off value provided by the manufacturer (14 pg/ml), the sensitivity of specificity of IGRA were 77.59% (180/232) and 76.47% (182/238), respectively (Table 2). The GBP5 assay and IGRA had comparable sensitivity for the diagnosis of PTB and EPTB, but the specificity of GBP5 was significantly lower than that of IGRA (p = 0.025). The majority of NTM patients (72.73%) were positive in the GBP5 assay, and only 18.18% of NTM patients were positive in IGRA (p = 0.030), while there was no significant difference in the positive rate observed between the GBP5 assay (31.28%) and IGRA (23.793%) in patients with OLD (p = 0.925). From these results, a positive predictive value (PPV) of 69.60% and a negative predictive value (NPV) of 75.71% were observed for the GBP5 assay, and a PPV of 76.27% and an NPV of 77.78% were observed for IGRA (Table 2).

### The combination of whole blood GBP5 protein levels and IGRA for aTB diagnosis

**Fig. 4:**
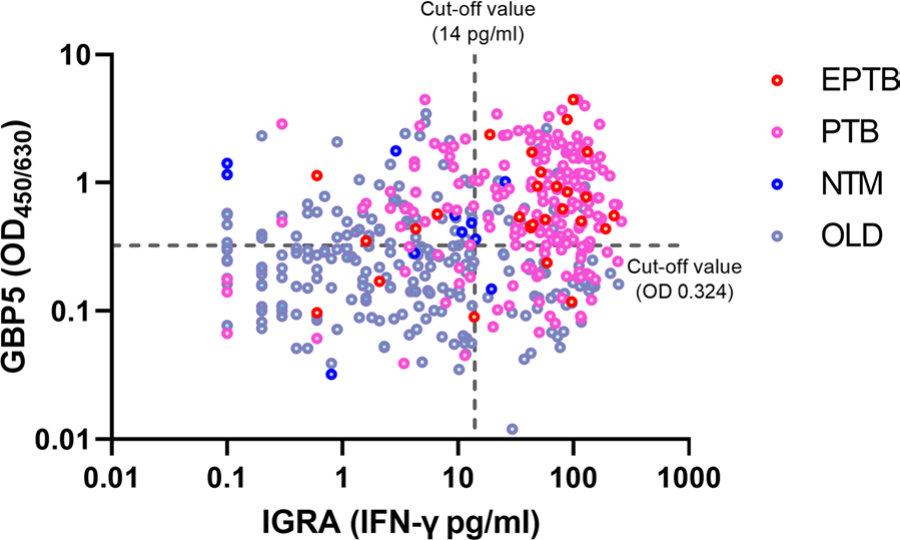
Scatterplot of GBP5 levels and IGRA results

When the scatter graph of GBP5 levels and IGRA results was plotted, no significant correlation was observed between them (r = 0.17) (Fig. 4). With the abovementioned cut-off values, 63.83% of all suspected patients (300/470) presented consistent results in the GBP5 assay and IGRA (163 with double positive results and 137 with double negative results), and 36.17% (170/470) had single positive results (73 with only IGRA positive results and 97 with only GBP5 positive results) (Additional file 2: Table S1). Among patients with double-positive results, 89.57% were finally diagnosed with aTB, while 87.59% of patients with double-negative results were diagnosed with non-TB. The 170 suspected patients with single positive results had only a 40.59% probability of being interpreted with aTB. When IGRA^+^/GBP5^+^ and IGRA^−^/GBP5^−^ were used as diagnostic criteria for aTB and non-aTB, respectively, 300 out of 470 suspected patients (63.83%) could be successfully diagnosed with an accuracy of 88.52% (266/300) (Additional file 2: Table S1). With these diagnostic criteria, a PPV of 89.57% (146/163) and an NPV of 87.59% (120/137) were achieved, although 36.17% of the total suspected patients with single positive results could not be certainly diagnosed.

## Discussion

Despite intensive efforts, the rapid and accurate diagnosis of aTB is still a challenge. It was recently reported that the mRNA levels of several genes and gene sets in whole blood could be new and practical biomarkers for aTB diagnosis [20, 21]. However, nucleic acid testing is difficult to widely utilize in the clinic in low-income countries with a high burden of tuberculosis due to its complex operation, high cost, and high requirements for personnel and facilities. In this study, an ELISA for detecting whole blood GBP5 protein was developed, and it was found that the levels of whole blood GBP5 protein have the potential to differentiate aTB from non-TB.

In recent years, with the in-depth study of tuberculosis, host blood transcriptional signatures and host response signatures have been reported to be different between aTB patients and LTBI patients, healthy controls, or patients with other lung diseases [22–24]. This year, in a prospective study comparing the aTB diagnostic accuracy of reported blood transcriptional signatures in real-world settings, Sweeney’s 3 signature genes manifested the best performance, with an area under the receiver operating characteristic curve (AUC) of 0.906 [25]. Among these 3 genes, Sweeney et al. also found that the blood transcriptional level of *GBP5* had the most significant mean difference between LTBIs and other lung diseases and aTB [17].*GBP5* is an interferon-inducible gene [26]. Although elevated whole blood transcriptional levels of *GBP5* in aTB have been previously reported [17, 18, 25], elevated whole blood levels of GBP5 protein in aTB were first reported in this study.GBP5 protein levels were increased in both the aTB groups, PTB and EPTB, without a significant difference (Fig. 2A), which indicates that the whole blood GBP5 protein levels resulting from aTB were not influenced by the organs/sites where tuberculosis lesions occurred. As an effector of the innate immune response, the whole blood levels of GBP5 were elevated in some non-TB patients, especially in the NTM group (Fig. 2A). The results indicate that NTM infection in the lung results in a robust expression of GBP5 protein in circulating blood cells.

No significant correlation between whole blood GBP5 protein levels and IGRA results was observed (Fig. 4). The GBP5 protein assay detects GBP5 protein levels in unstimulated whole blood samples and its elevation results from ongoing diseases, while IGRA tests samples stimulated with MTB-specific antigens and the positive results indicate MTB infection, including aTB and LTBI. Based on the difference between the GBP5 assay and IGRA, the combination of both results of the two assays presented an improved performance for aTB diagnosis in a major proportion of suspected patients (Additional file 2: Table S1). Out of a total of 470 suspected patients, 300 (63.83%) had consistent results in the GBP5 assay and IGRA (both positive and both negative), and aTB may be diagnosed with an accuracy of 88.52% (266/300) in these patients.

*GBP5* is a representative gene of reported transcriptional signatures that are diagnostic for aTB. This study validated that detecting GBP5 protein levels also has diagnostic potential for aTB as the testing of GBP5 mRNA levels in whole blood [27, 28]. Assays for detecting the protein levels of other genes belonging to these transcriptional signatures can be developed and assessed. It is promising to develop a protein assay or a combination of these protein assays with high performance for aTB diagnosis. The 3 signature genes discovered by Sweeney, including *GBP5*, *DUSP3* and *KLF2*, have been reported to have outstanding performance for aTB diagnosis in real-world settings [18, 25]. Therefore, it can be speculated that the combination of GBP5, DUSP3 and KLF2 protein levels in whole blood will also have good aTB diagnostic performance.

The major limitation in this study is that a quantitative GBP5 protein assay was not developed and used. Due to the lack of quantitative standards, the OD values were directly used as the GBP5 protein levels, which would result in low reproducibility in different laboratories and high variation in different microplates. To further evaluate the assay’s performance for aTB diagnosis in the future, it is a priority to establish quantitative standards. In addition, the rate of vaccination with the BCG vaccine was not included in this study, which may have an impact on the results. Follow-up studies will explore the diagnostic value of GBP5 with or without BCG vaccination.

In conclusion, GBP5 protein in whole blood is a potential biomarker for differentiating aTB from non-TB. The whole blood GBP5 protein assay has similar performance for aTB diagnosis as IGRA, and the combination of the GBP5 assay and IGRA results in approximately 90% accuracy for diagnosing aTB in more than half of the suspected patients. A quantitative GBP5 protein assay should be developed, and its clinical value needs to be further studied. This study provides an essential idea for realizing the clinical application of the findings of whole blood transcriptomics by immunological methods instead of nucleic acid testing.

## Supplementary Information

**Additional file 1: Fig S1. **Protein profile of GBP5 protein.

**Additional file2: Table S1. **The distributions of patients with different GBP5 and IGRA results.

**Additional file 3: Fig S2.** Reactivity of GBP5 protein assay developed with the mAb pair of 7G9 and 9A9.

## Data Availability

The datasets used and/or analysed during the current study are available from the corresponding author on reasonable request.
