# Supplementary material for: Whole blood GBP5 protein levels in patients with and without active tuberculosis
Source: BMC Infect Dis. 2022 Apr 3;22:328. doi: 10.1186/s12879-022-07214-8 (PMC8976871; doi:10.1186/s12879-022-07214-8)
Supplement: Supplementary file 1 — Additional file 1: Fig S1. Protein profile of GBP5 protein. [file 12879_2022_7214_MOESM1_ESM.pptx]

## Slide 1
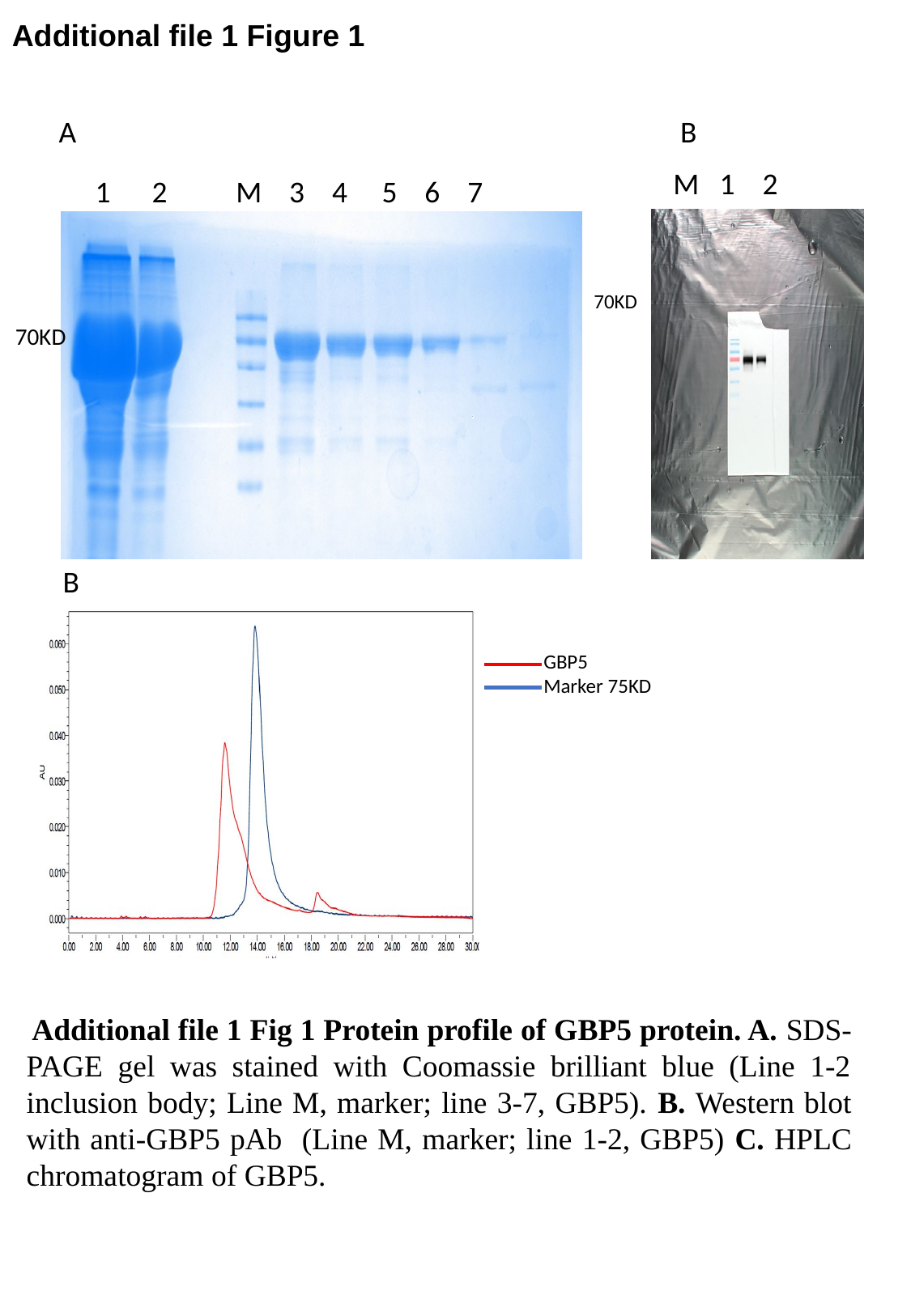

Additional file 1 Figure 1
A
B
M 1 2
1 2 M 3 4 5 6 7
70KD
70KD
B
GBP5
Marker 75KD
 Additional file 1 Fig 1 Protein profile of GBP5 protein. A. SDS-PAGE gel was stained with Coomassie brilliant blue (Line 1-2 inclusion body; Line M, marker; line 3-7, GBP5). B. Western blot with anti-GBP5 pAb (Line M, marker; line 1-2, GBP5) C. HPLC chromatogram of GBP5.
