# Supplementary material for: Whole blood GBP5 protein levels in patients with and without active tuberculosis
Source: BMC Infect Dis. 2022 Apr 3;22:328. doi: 10.1186/s12879-022-07214-8 (PMC8976871; doi:10.1186/s12879-022-07214-8)
Supplement: Supplementary file 2 — Additional file2: Table S1. The distributions of patients with different GBP5 and IGRA results. [file 12879_2022_7214_MOESM2_ESM.docx]

**Additional file 2: Table S1 The distributions of patients with different GBP5 and IGRA results**

|  | IGRA+ GBP5+ | IGRA+ GBP5- | IGRA- GBP5+ | IGRA- GBP5- |
| --- | --- | --- | --- | --- |
| Total | 163 (34.68%) | 73 (15.53%) | 97 (20.64%) | 137 (29.15%) |
| aTB | 146 (62.93%) | 34 (14.66%) | 35 (15.09%) | 17 (7.32%) |
| PTB | 128 (62.75%) | 32 (15.69%) | 30 (14.71%) | 14 (6.86%) |
| EPTB | 18 (64.29%) | 2 (7.14%) | 5 (17.85%) | 3 (10.71%) |
| non-TB | 17 (7.14%) | 39 (16.39%) | 62 (26.05%) | 120 (50.42%) |
| NTM | 1 (9.09%) | 1 (9.09%) | 7 (63.64%) | 2 (18.18%) |
| OLD | 16 (7.05%) | 38 (16.74%) | 55 (24.23%) | 118 (51.98%) |
|  |  |  |  |  |
| Sensitivity  (95% CI) | 62.93%  (56.62%-69.01%) |  |  | / |
| Specificity  (95% CI) | / |  |  | 50.42%  (44.13%-56.78%) |
| PPV  (95% CI) | 89.57%  (84.22%-93.59%) |  |  | / |
| NPV  (95% CI) | / |  |  | 87.59%  (81.31%-92.42) |

Abbreviations: IGRA+ GBP5+: Individual with positive GBP5 and interferon gamma release assay result; IGRA+ GBP5-, Individual with positive interferon gamma release assay and negative GBP5 result; IGRA- GBP5+, Individual with negative interferon gamma release assay and positive GBP5 result; IGRA- GBP5-, Individual with negative GBP5 and interferon gamma release assay result; aTB, active tuberculosis; PTB, pulmonary tuberculosis; EPTB, extrapulmonary tuberculosis; non-TB, non-tuberculosis other lung diseases; NTM, non-tuberculosis mycobacterium; OLD, other lung disease; PPV, positive predictive value; NPV, negative predictive value.
