# Supplementary material for: Whole blood GBP5 protein levels in patients with and without active tuberculosis
Source: BMC Infect Dis. 2022 Apr 3;22:328. doi: 10.1186/s12879-022-07214-8 (PMC8976871; doi:10.1186/s12879-022-07214-8)
Supplement: Supplementary file 3 — Additional file 3: Fig S2. Reactivity of GBP5 protein assay developed with the mAb pair of 7G9 and 9A9. [file 12879_2022_7214_MOESM3_ESM.pptx]

## Slide 1
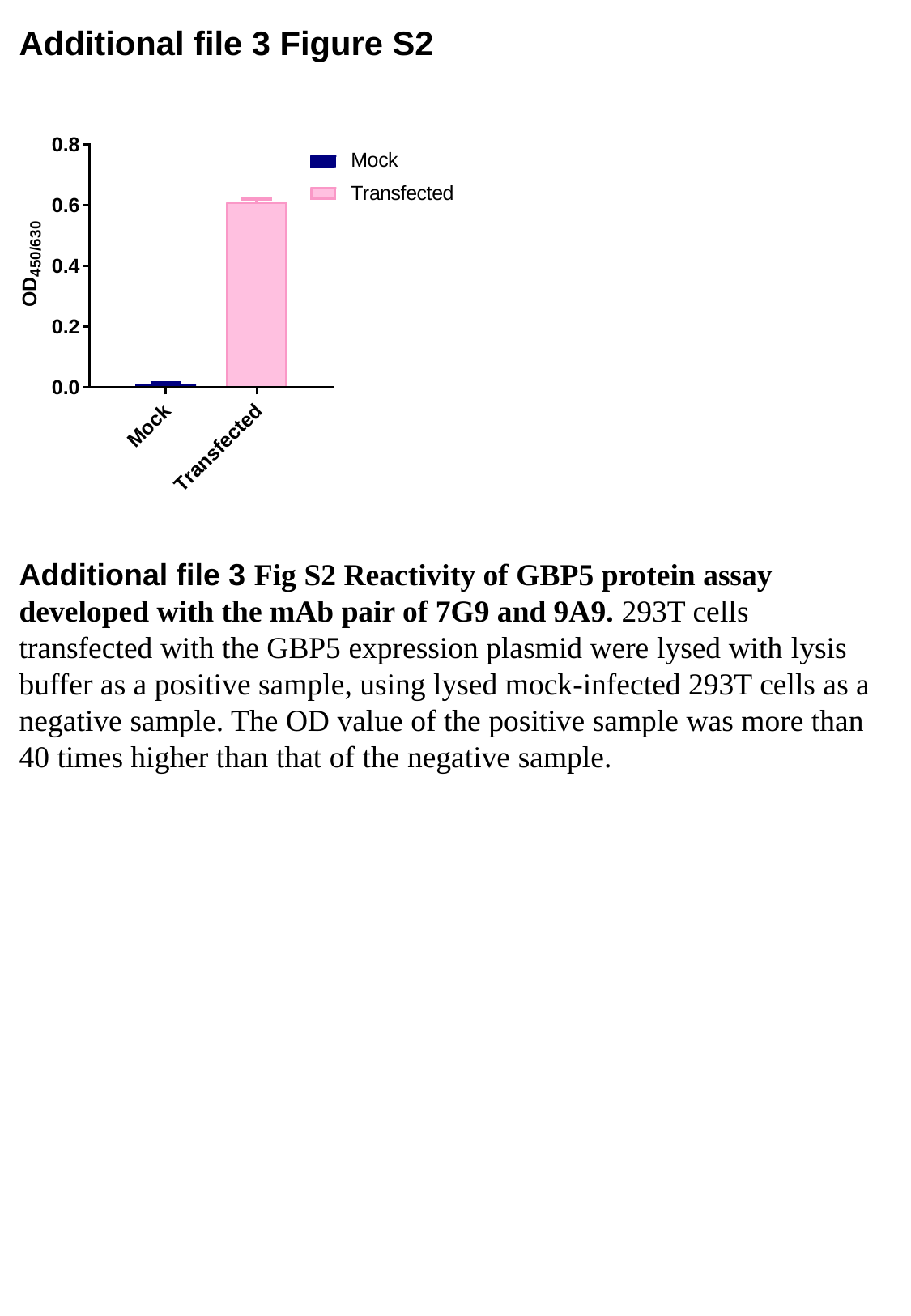

Additional file 3 Figure S2
Additional file 3 Fig S2 Reactivity of GBP5 protein assay developed with the mAb pair of 7G9 and 9A9. 293T cells transfected with the GBP5 expression plasmid were lysed with lysis buffer as a positive sample, using lysed mock-infected 293T cells as a negative sample. The OD value of the positive sample was more than 40 times higher than that of the negative sample.
